# Description of Two Species of Early Branching Dinoflagellates, *Psammosa pacifica* n. g., n. sp. and *P. atlantica* n. sp

**DOI:** 10.1371/journal.pone.0034900

**Published:** 2012-06-18

**Authors:** Noriko Okamoto, Aleš Horák, Patrick J. Keeling

**Affiliations:** 1 Department of Botany, University of British Columbia, Vancouver, British Columbia, Canada; 2 Laboratory of Molecular Taxonomy, Institute of Parasitology, Biology Centre of Academy of Sciences of the Czech Republic, České Budějovice, The Czech Republic; 3 Faculty of Sciences, Department of Evolutionary Protistology, University of South Bohemia, České Budějovice, The Czech Republic; Université Paris Sud, France

## Abstract

In alveolate evolution, dinoflagellates have developed many unique features, including the cell that has epicone and hypocone, the undulating transverse flagellum. However, it remains unclear how these features evolved. The early branching dinoflagellates so far investigated such as *Hematodinium*, *Amoebophrya* and *Oxyrrhis marina* differ in many ways from of core dinoflagellates, or dinokaryotes. Except those handful of well studied taxa, the vast majority of early branching dinoflagellates are known only by environmental sequences, and remain enigmatic. In this study we describe two new species of the early branching dinoflagellates, *Psammosa pacifica* n. g., n. sp. and *P. atlantica* n. sp. from marine intertidal sandy beach. Molecular phylogeny of the small subunit (SSU) ribosomal RNA and Hsp90 gene places *Psammosa* spp. as an early branch among the dinoflagellates. Morphologically (1) they lack the typical dinoflagellate epicone–hypocone structure, and (2) undulation in either flagella. Instead they display a mosaïc of dinokaryotes traits, i.e. (3) presence of bi-partite trychocysts; *Oxyrrhis marina*–like traits, i.e. (4) presence of flagellar hairs, (5) presence of two-dimensional cobweb scales ornamenting both flagella (6) transversal cell division; a trait shared with some syndineansand *Parvilucifera* spp. i.e. (7) a nucleus with a conspicuous nucleolus and condensed chromatin distributed beneath the nuclear envelope; as well as *Perkinsus marinus* -like features i.e. (8) separate ventral grooves where flagella emerge and (9) lacking dinoflagellate-type undulating flagellum. Notably *Psammosa* retains an apical complex structure, which is shared between perkinsids, colpodellids, chromerids and apicomplexans, but is not found in dinokaryotic dinoflagellates.

## Introduction

Alveolates are a major eukaryotic assemblage that includes three large and well-studied lineages, apicomplexans, dinoflagellates, and ciliates [1], [2]. Each of these lineages has developed remarkable and complex innovations, sometimes taken to extremes, and alveolate evolution has consequently been a topic of considerable interest. In the case of apicomplexans and dinoflagellates, reconstructing the evolution of their unusual characteristics has been aided tremendously by the discovery of deep-branching relatives of both lineages. Interestingly in the case of the non-photosynthetic apicomplexans, where recently discovered photosynthetic relatives had led to several breakthroughs [3], [4], [5], [6].

About half of the described species of dinoflagellates are photosynthetic, and the rest are grazers or parasites [7]. The well-studied “core” dinoflagellates, or the dinokaryotes, share distinctive features, i.e. dinokaryon (nucleus with permanently condensed chromosomes), the motile cell consists of two distinctive parts named epicone and hypocone, and an undulating transverse flagellum and a straight longitudinal flagellum [7]. Moreover, a number of unusual molecular innovations have also been found to be universal among dinokaryotes, including unusual organisation of organelle genomes [8], [9], [10], [11], [12], [13], [14], and messenger RNAs with spliced leaders [15], [16], [17]. How these features evolved and relate to the evolution of their apicomplexan relatives is, however, not clear from the study of dinokaryotes alone: to understand the evolution of such features, a greater knowledge of the whole dinoflagellate diversity is required.

Environmental sequence data reveal that there is substantial unexplored diversity within the dinokaryotes but even more so among the basal lineages of dinoflagellates, many of which remain unknown at the cellular level [18], [19], [20], [21], [22], [23], [24], [25], [26], [27], [28], [29], [30], [31]. Those basal lineages were referred to as marine alveolate groups (MAG) in the early reports, and are now confirmed to include a handful of parasitic dinoflagellate groups historically called syndineans [32], [33], [34], [35], [36], [37], [38], [39].

Syndineans are groups of intracellular parasitic dinoflagellates characterized by the absense of dinokaryotes or theca (cell wall of the “armoured” dinoflagellaltes), the presence of alveoli vesicle underneath the plasma membrane. The motile stage of syndineans has epicone-hypocone architecture and laterally inserted flagella in most known cases [40]. Syndineans, as originally described, are polyphyletic, including some dinokaryotes such as *Blastodinium* spp. and others that branch paraphyletically at or near the base of dinokaryotes. These basal syndinians currently include seven genera, namely, *Syndinium*, *Hematodinium*, *Amoebophrya*, *Euduboscquella*, *Ichthyodinium*, *Ellobiopsis* and *Thalassomyces*. Molecular phylogeny revealed that *Syndinium*, *Hematodinium* and *Amoebophrya* are part of MAG II [24], [33], [36], [41], [42], [43], [44]; and that *Euduboscquella* and *Ichthyodinium* are part of MAG I [34], [37], [39], [45], [46]. The ellobiopsids, consisting of *Ellobiopsis* and *Thalassomyces,* form an independent and fast evolving lineage that does not belong to either of these major clades [32], [38]. Although the branching order of these early diverging lineages and the dinokaryotes has not been satisfactorily resolved, their early divergence from the ancestor of dinokaryotes is generally supported by molecular phylogenetic data and “syndinean-like” nuclear morphology *sensu* Leander & Hoppenrath [47] i.e., centrally located nucleolus with peripherally condensed chromatins that are also found in some perkinsids and colpodellids, as well as the intra nuclear spindle during nucleokinesis, which are shared with the apicomplexans [7], [40], [43], [48], [49]. Those basal dinoflagellates may hold the keys to understanding many aspects of dinoflagellate early evolution. However, the primary challenge is that we don’t have the information of MAG I and II at cellular level, except those eight genera. Secondarily, even among those parasitic genera, there’re limited cases of ultrastructural studies on the flagellate stage (*Ichtyodinium chabelardi* [44] in MAG I, *Hematodinium* sp. [50] and *Amoebophrya* spp. in MAG II [51], [52], [53]). This burdens a direct comparison between the dinokaryotes, perkinsids, or the more distantly related apicomplexans. In addition to MAG I and II, there are several taxa that are argued to be early diverging dinoflagellates such as *Oxyrrhis marina*, though their phylogenetic positions are yet to be determined.

In this study, we report a newly discovered free-living flagellates that branches among the early-diverging dinoflagellates, *Psammosa pacifica* n. g., n. sp. and *P. atlantica* n. sp. We discovered *P. pacifica* from Boundary Bay, British Columbia and *P. atlantica* from Blomidon Beach, Bay of Fundy, Nova Scotia, Canada. *Psammosa* cells are dorsoventrally compressed barley shape. The cell has a protrusion in the middle of dorsal face and grooves on the both side of the protrusion, where a shorter anterior and longer posterior flagellum separately emerge from distinct grooves. Cell division occurs along the transversal plane. *Psammosa* is a predator, feeding on other eukaryotes such as a heterotrophic stramenopile flagellates (*P. pacifica*) or diatoms (*P. atlantica*).

Only flagellate cells are observed for both species of *Psammosa*, which proliferate via transversal fission as is shown in *O. marina*. Ultrastructural observation of *P. pacifica* revealed that it possesses bipartite trichocysts and a syndinian-like nucleus, as found in the dinokaryotes, perkinsus and some colpodellids; a flagellar transition region with an inclusion body as is found in some syndineans and perkinsids; two-dimensional flagellar scales and flagellar hairs as found in *O. marina*; and most remarkably an apical complex with pseudoconoid such as that found in perkinsids and colpodellids.

Small subunit (SSU) ribosomal DNA and heat shock protein 90 (Hsp90) sequence were characterised from *Psammosa*, and phylogenetic analysis, together with a unique insertion deletion character in Hsp90 [54], show that they form an independent lineage branching early in the tree of dinoflagellates. Altogether, we concluded that *Psammosa* represents the earliest lineage of dinoflagellates known to date, and as such has the potential to provide many insights into early alveolate evolution.

## Results

### Taxonomic Summary

#### Assignment

Eukaryota; Chromoalveolata; Alveolata; Dinozoa.

*Psammosa* n. g. N. Okamoto, A. Horák and Keeling, 2012.

urn:lsid:zoobank.org:act:6C90BBAE-A2F5-4F1A-ABA3-DD77645AF331.

#### Diagnosis

The cell is biflagellate, dorsoventrally compressed barley shape with the round anterior end and acute posterior end, with a kink on the left ventral contour. It has a subapical diagonal ridge on the ventral face dividing anterior-left section and posterior-right section of the cell. The ventral side of the anterior-left section is concave towards the margin to make a wide groove on the right side of the ridge, where the anterior flagellum is inserted. The posterior-right section has a longitudinal depression in the middle, where the posterior flagellum is inserted. The cell is devoid of body scale. Both flagella bear two dimensional scales and mastigonemes. The cell contains a refractile body in the posterior section. The cell is colorless and devoid of any visible evidence of plastid. The cell proliferates by binary fission along the transverse plane at the margin of the anterior-left section and posterior-right section where the anterior flagellum emerge. The cell often rests on the bottom surface with the periodically beating posterior flagellum. The cell swim with rotation in the water column; while on the bottom surface, it swims in a tight circle pivoting around the cell apex without rotation. Both the anterior and the posterior flagella bears simple mastigonemes and two dimensional cobweb-shape flagellar scales.

#### Type species

*Psammosa pacifica* N. Okamoto, A. Horák and Keeling, 2011.

#### Etimology

*Psammosa* is derived from the greek psammon, meaning sand, the material from which both species of *Psammosa* were isolated.

*Psammosa pacifica* n. sp. N. Okamoto, A. Horák and Keeling, 2011.

urn:lsid:zoobank.org:act:E3F62250-F13C-4E2D-B7AB-7CC83F67C7D4.

#### Diagnosis

Cell is 7–8 µm in length and 4–5 µm in width, dorsoventrally compressed barley shape with a round anterior end and an acute posterior end. The posterior end of the anterior-right section and the posterior left section meet at c.a. two third of the entire cell length. Apical pore locates at the anterior cell apex. Cell is eukaryvore and feeds on *Spumella* sp. (Chrysophyceae, Stramenopiles). Marine interstitious.

#### Type locality

The cells were collected from the interstitious water in the intertidal sandy beach at Boundary Bay, British Columbia, Canada (49.0086°N; −123.0228°W).

#### Type figure

Fig. 1a.

**Figure 1:**
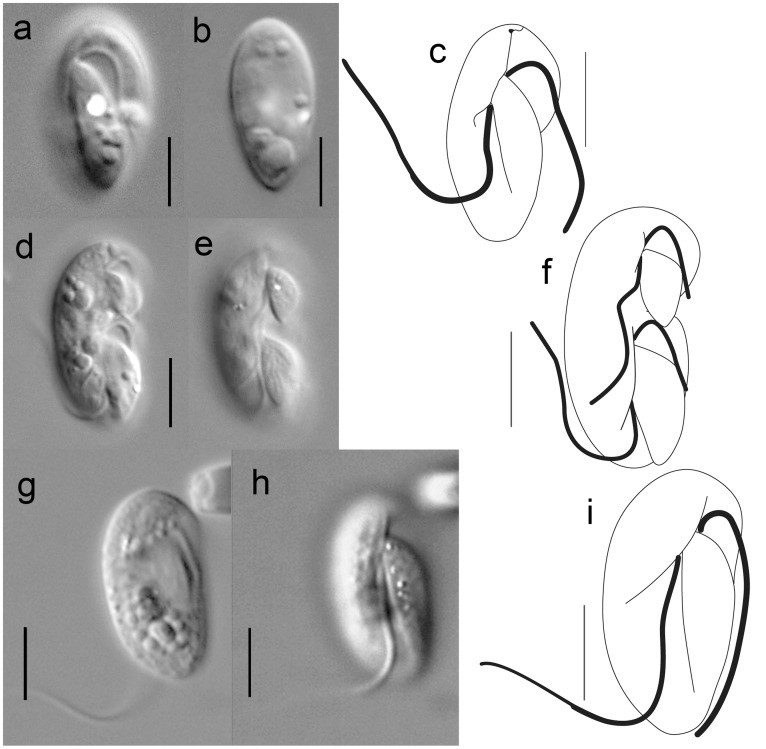
General morphology of *Psammosa pacifica* and P. atlantica. **a–c**. *P. pacifica*; **d–e**. dividing cell of *P. pacifica*. **g–i**. *P. atlantica.* Scales  = 5 µm.

#### Type sequence

Partial small subunit ribosomal RNA gene of *P. pacifica*: JN873311.

#### Type specimen

TEM block deposited in Marine Invertebrate Collection, Beaty Biodiversity Museum, UBC: MI-PR110.

#### Etymology

Epithet refers to the ocean that type locality situates.

### General Morphology of *Psammosa pacifica* n. sp

Cell is 7–8 µm in length and 4–5 µm in width, dorsoventrally compressed barley shape with a round anterior end and an acute posterior end. The posterior end of the anterior-right section and the posterior left section meet at c.a. two third of the entire cell length. The cell is a eukaryovore, feeding on small flagellates such as *Spumella* sp. Prey is taken up at the apical end of the cell without changing the cell shape as would be seen in phagocytosis (Figure 1a).

#### Cell division of *P. pacifica*

Cells undergo division transversally along the boundary between the anterior right and the posterior left sections of the cell (Fig. 1c–d). Flagellar duplication initiates prior to cell fission. Increasing numbers of the dividing cells are observed towards the end of the dark period under culture conditions.

#### Swimming behaviour of *P. pacifica*

The cell swims with rotation in the water column; while on the bottom surface, it swims in a tight circle pivoting around the cell apex without rotation (Movie S1, S2, S3). Cells are sometimes observed resting on the surface of culture flasks with the posterior flagellum beating periodically.

#### Surface morphology of *P. pacifica*

**Figure 2:**
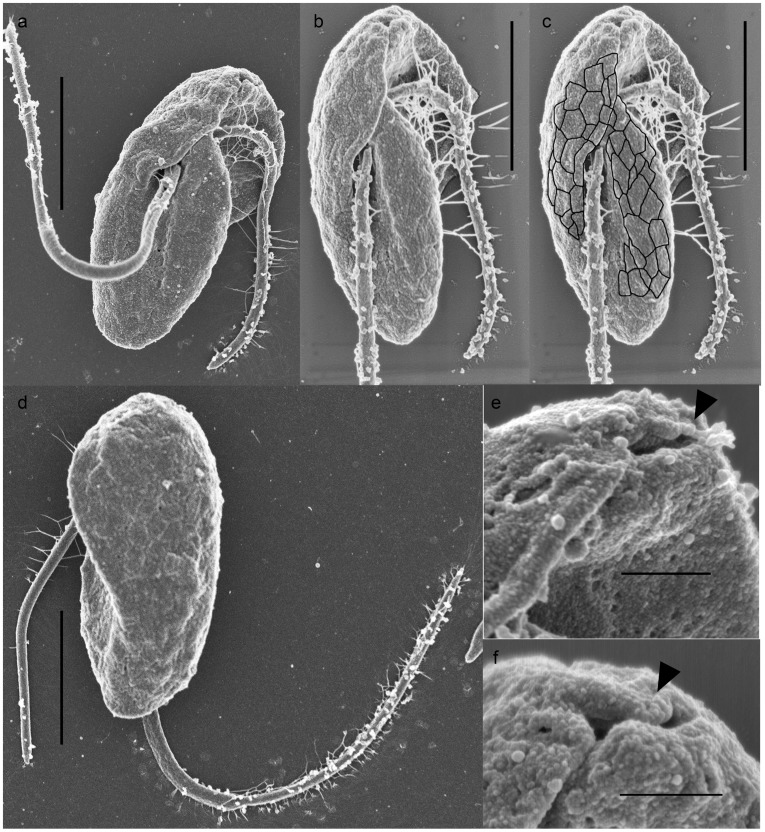
Surface morphology of *Psammosa pacifica.* **a–c**. Ventral view of *P. pacifica*. **b** and **c** is the same cell. The poligonal margins of the alveoli vesicles are indicated to facilitate the visualization. **d**. Dorsal view of *P. pacifica*. **e–f**. Close up view of the apical pore. Scales  = 3 µm in **a–d**; 500 nm in **e–f**.

The cell lacks body scales. SEM preparation caused a slight shrinkage of the cell and revealed the pattern of alveoli beneath the cell membrane (Fig. 2a–d). The alveoli are arranged in polygonal pattern over the entire surface of the cell (Fig. 2c). Closer observation under SEM revealed that a small lobe of cytoplasm is situated on the right side of the anterior and posterior flagellar insertions (Fig. 2a–b). The anterior end of the lobe terminates with a pore (an opening of a narrow invagination near the cell apex) (Fig. 2e–f), which coincides with the point of contact with the food/prey cell (not shown).

**Figure 3:**
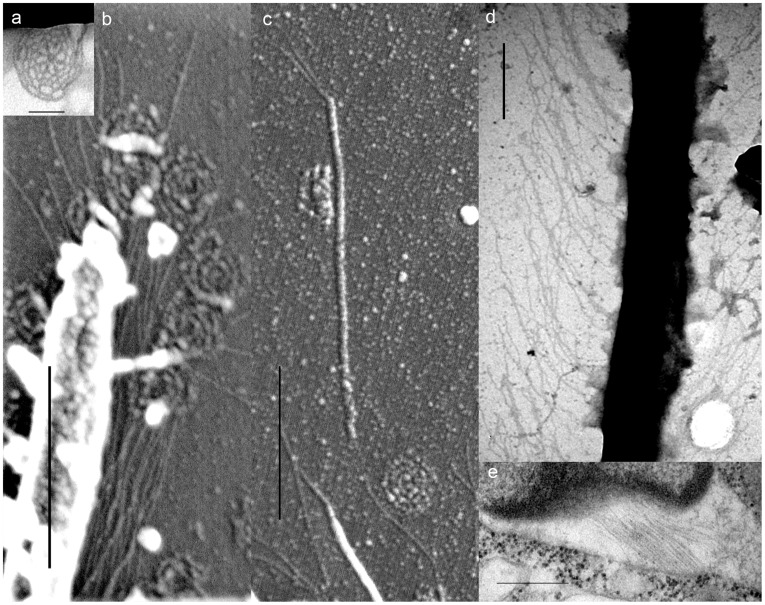
Flagellar appendages of *Psammosa pacifica.* **a**. A flagellar scale showing cob-web pattern prepared by whole-mount method. b. A close up view of flagellum to show the flagellar hairs (mastigonema) and scales. **c**. A detatched flagellar hair (mastigoneme) showing the shaft and fine hairs. **d**. The flagellar hairs (mastigonema) on a flagellum prepared by whole-mount method. **e**.The compartment between the inner and the outer nuclear envelops contains fine hairs similar to mastigonema on flagella. Scales  = 100 nm in **a**; 500 nm in **b**–**e**.

### Flagellar Scales, Mastigonemes and Flagellar Transition Region of *P. pacifica*

The cell has short anterior and long posterior flagella (Fig. 1a–b, 2a, d). The anterior flagellum is inserted transversally and no undulation of either flagellum is observed. When the cell is resting on the bottom of the culture vessel, the posterior flagellum beats periodically while the anterior flagellum shows little movement.

The posterior flagellum is ca. 380 µm in diameter, along the proximal half of the length, then bluntly reduce to ca. 250 µm in diameter along the distal half. The anterior flagella is consistent in its diameter, ca. 250 µm.

Both flagella are covered by elliptical scales along their entire length (Fig. 2a–d, 3a–c). Preparation for SEM and TEM forced the scales to detach from flagella. The scale is ca. 210 nm in longer diameter and ca. 180 nm in shorter diameter, with a cobweb-like patterns consisting of 8 spokes radiating from the centre, and the 4–5 rings bridging the spokes (Fig. 3a–b). It is unclear in which subcellular compartment the scales are produced.

In addition to the flagellar scales, the entire anterior flagellum and the distal half of the posterior flagellum bears bipartite hairs composed of shaft and with 1–2 terminal hair(s) (Fig. 3b–d). Similar hair structures are observed between the inner and the outer nuclear membranes (Fig. 3e).

**Figure 4:**
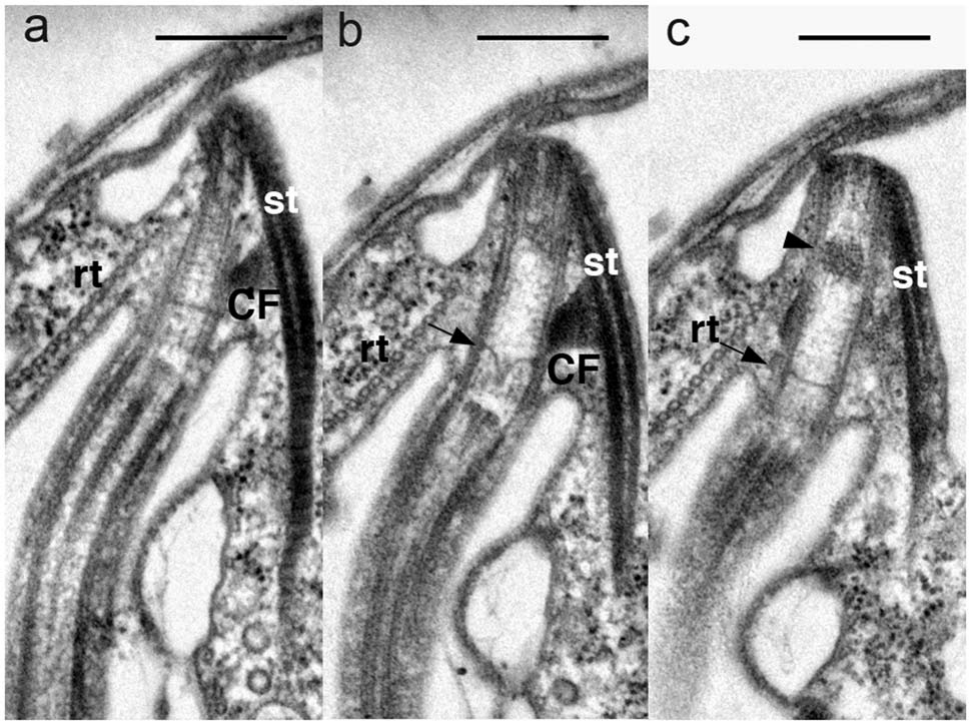
Flagellar transition region of *P. pacifica*. a–c . A series of sections of the posterior flagellum. **a**. The posterior basal body is associated with striated fibers (st), connecting fibers (CF) and microtubular root (rt). **b**. A terminal plate (arrow) acutely protruding towards the distal end. **c**. A dark stained inclusion body (arrowhead) is situated proximal side of the terminal plate. Scales  = 500 nm.

In the flagellar transition region, the central pair of microtubules terminate above the basal plate (Fig. 4a–c). Electron dense material was present on the outside of the central pair at termination. The centre of the basal plate is curved sharply toward the distal end of the transition region (Fig. 4b). Beneath the basal plate, lies an electron dense black globule in the posterior flagellar basal body (Fig. 4c).

### Intracellular Ultrastructure of *P. pacifica*

**Figure 5:**
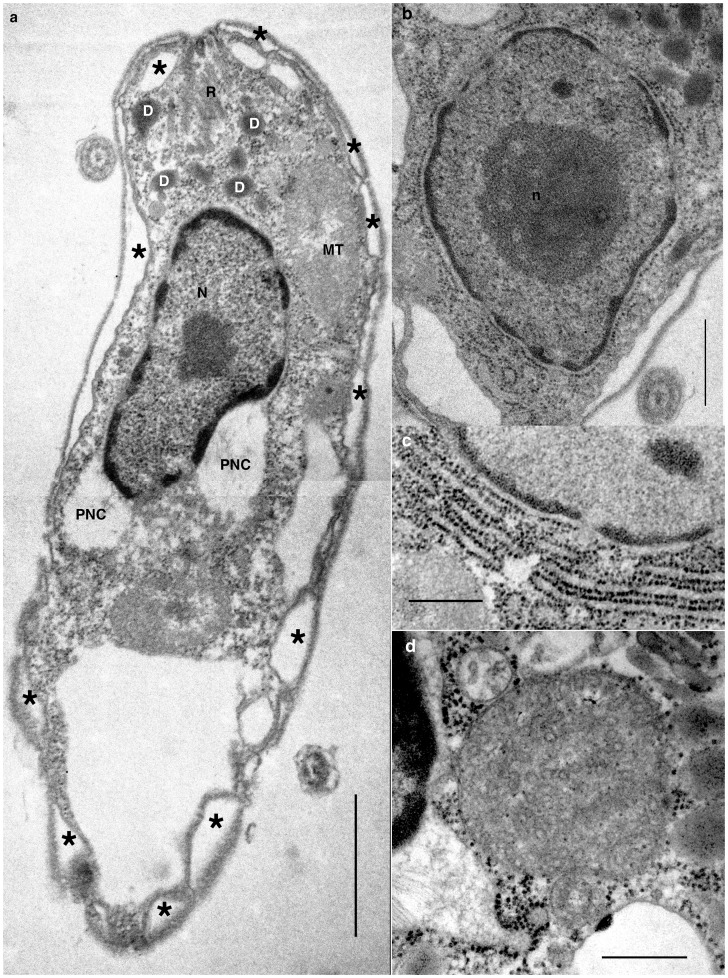
Internal structure of *P. pacifica.* **a**. longitudinal section of *P. pacifica*. **b**. Nuclear contents are condensed on periferal. nucleolus is situated in the middle of the nucleus. **c**. rough endoplasmic reticulum is connected to the ourter nuclear envelop. **d**. Profile of mitochondrion shows the tubular cristae. D  =  dark stained vesicles; PNC  =  perinuclear compartment; MT  =  mitochondrion; N  =  nucleus; n  =  nucleolus; R  =  rhoptries; astarisks  =  alveolar vesicles. Scales  = 1 µm in **a**; 500 nm in **b–d**.

The nucleus is situated in the mid-anterior region of the cell (Fig. 5a). The nucleus is not a dinokaryon, but rather is a syndinian-like [47], with dark stained materials lining the inner surface of the inner nuclear membrane and a spherical nucleolus situated in the centre of nucleus (Fig. 5a–b). The space between the outer and inner nuclear membranes is swollen and has fibrous material viable (Fig. 5a). Part of the outer nuclear membrane is studded with ribosomes and is associated with the rough endoplasmic reticulum (ER) (Fig. 5c). The Golgi body was not observed. Alveolar vesicles of various size are situated underneath of cell membrane and are found throughout the entire cell surface, except at the grooves where flagella are inserted. The alveoli were not observed to contain electron dense fibrils or theca (Fig 5a; asterisks).

The Mitochondrion is often found anterior to the nucleus. Its morphology is as expected for an alveolate, with clearly tubular cristae (Fig 5d).

**Figure 6:**
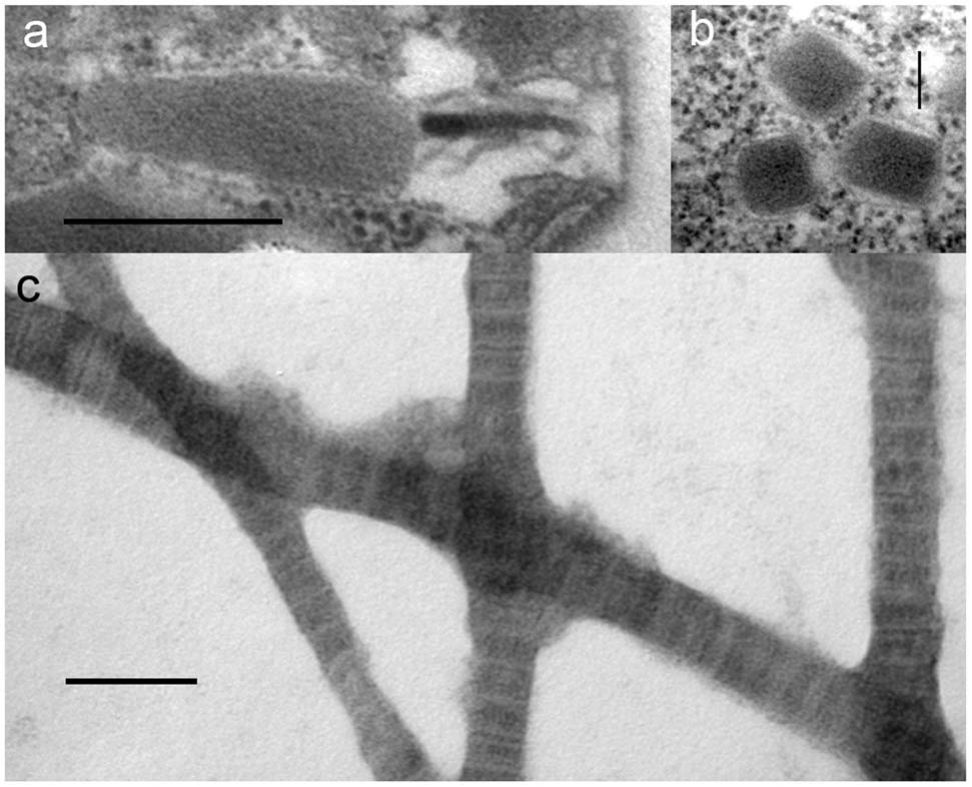
Trichocyst of *P. pacifica.* **a**. Longitudinal section of a trichocysts composed with dark stained shaft and lightly stained rod contained in a single membrane vesicle. **b**. transverse section of trichocysts showing square profile of the rod. **c**. Discharged trichocysts showing striated ribbon-like structure. Scales  = 500 nm in **a**; 100 nm in **b–c**.

Bipartite trichocysts enclosed in a single membrane are present beneath the entire cell surface (Fig. 6a–b). A trichocyst is composed of dark stained rod that has square profile (Fig. 6b), and lightly stained head of ca. 1 µm in length (Fig. 6a). In response to stimulation, such as chemical fixation of whole mounts for transmission electron microscopy, trichocysts discharge a ribbon-like structure (Fig. 6c) with a maximum width of ca. 10–17 nm and striations consisting of ca. 6 nm of dark and ca. 2 nm of light in an alternating pattern, in some views with an additional narrow dark band within the light band.

**Figure 7:**
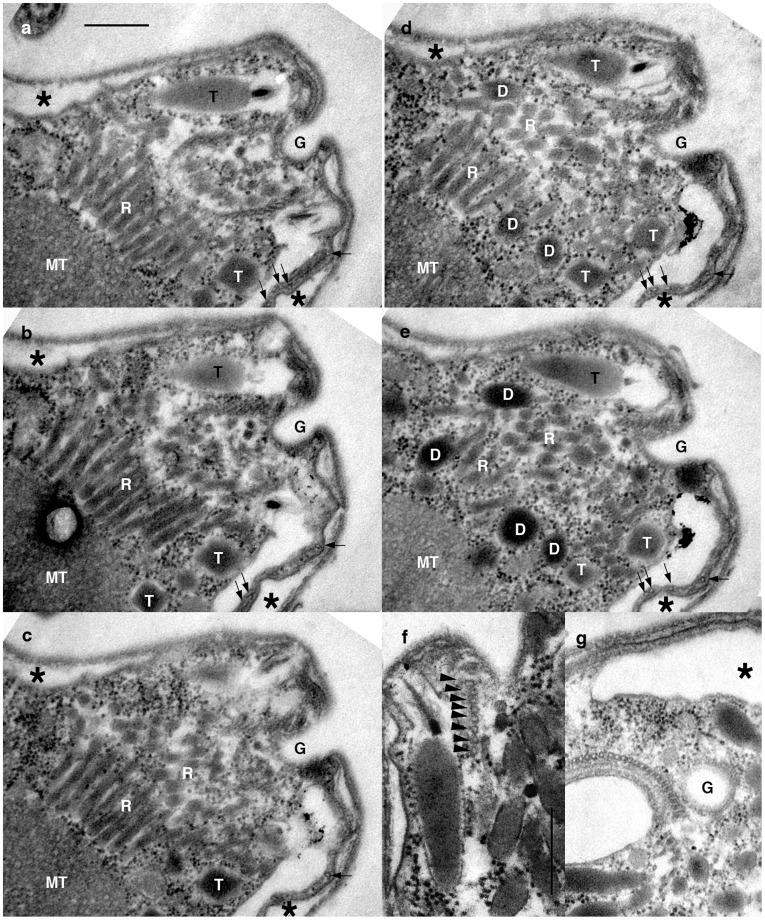
The apical complex of *P. pacifica*. **a–e**. A series of longitudinal section along dorsoventral axis of the apical complex showing 8 microtubules of pseudoconoid (arrowheads) associated with elongated rhomboid structure of rhoptories (R) near a gullet (G). f. Profile of pseudoconoid microtubules. **g.** A longitudinal section along sinistrodextral axis of the apical complex showing a cluster of rhoptries, dark stained vesicles (D) and gullet (G). MT  =  mitochondrion; N  =  nucleus; T  =  trichocyst; Asterisk  =  Alveoli vesicles; Double arrowheads  =  cortical microtubules underneath the alveoli vesicles. Scales  = 500 nm.

An apical complex is present at the anterior end of the cell (Fig. 7a). The complex consists of a pseudoconoid and two electron dense vesicles: longitudinally aligned rod-shaped rhoptries-like vesicles arranged in parallel to each other, and large spherical bodies situated posterior to the rhoptries (Fig. 7a–b). The pseudoconoid is composed of eight microtubules arranged in a slight curve convex toward the ventral side of the cell (Fig. 7a; arrowheads). The apical complex is associated with a gullet (Fig. 7b; G) that leads to the apical pore (Fig. 2e–f, 7a).

*Psammosa atlantica* n. sp. N. Okamoto, A. Horák and Keeling, 2012.

urn:lsid:zoobank.org:act:619DEF6-42D3-46A1-8BDB-D04C721BE424.

#### Diagnosis

Cell is 10–13 µm in length and 5–10 µm in width, dorsoventrally compressed barley shape with a round anterior end and an truncated posterior end. The posterior end of the anterior-right section and the posterior left section meet at c.a. one third of the entire cell length. Cell is eukaryvore and feeds on *Navicula* sp. (PRA-314, ATCC, VA) (Bacillariophyceae, Stramenopiles). Marine interstitious.

#### Type locality

The cells were collected from the interstitious water in the intertidal sandy beach at Blomidon Beach in the Bay of Fundy, Nova Scotia, Canada (45.25580°N; −64.34907°W).

#### Type figure

Fig. 1g.

#### Type sequence

Partial small subunit ribosomal RNA gene of *P. atlantica*: JN873310.

#### Type specimen

SEM deposited in Marine INvertebrate Collection, Beaty Biodiversity Museum, UBC:MI-PR111.

#### Etymology

Epithet refers to the ocean that type locality situate.

### General Behaviour of *Psammosa atlantica* n. sp

Cells are often observed resting on the bottom surface of culture vessel and seldom swim up towards mid to top layer of water column. Cells started swimming vigorously upon addition of ubiquinone-ethanol solution. The cell is eukaryovore feeding on a small pennate diatom *Navicula* sp. The prey uptake was not directly observed, though the frustule of the prey was never observed within *P. atlantica* cells.

### Surface Morphology and Flagellar Appendages of *P. atlantica*

**Figure 8:**
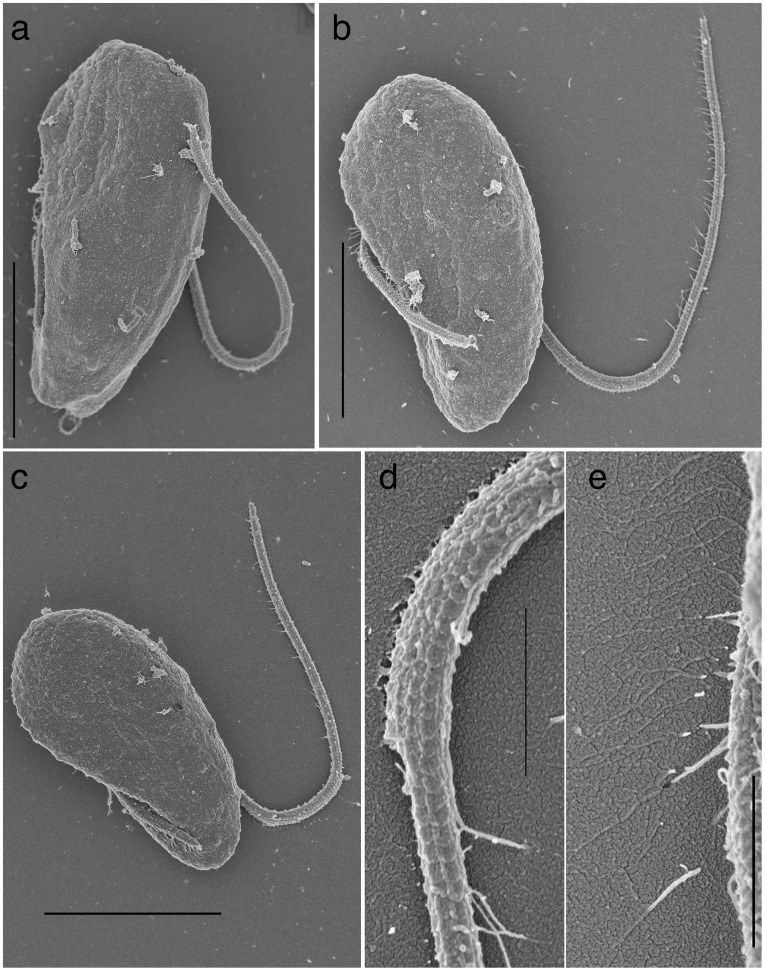
Surface morphology of *P. atlantica*. **a–c**. dorsal view. **d**. A close up view of the middle of the posterior flagellar showing a blunt reduction in the thickness. Rows of flagellar scales covers the entire surface of the anterior and the posterior flagella in an overlapping manner. The flagellar hairs (mastigonema) are present, though the special association with the scales is not clear **e**. A close up view of the anterior flagellar showing flagellar scales and hairs (mastigonema). Scales  = 5 µm in a–c; 500 nm in d–e.

The cell lacks body scales. The pattern of alveoli vesicles was visible after a slight shrinkage during SEM preparation (Fig. 8a–c). The alveoli are arranged in polygonal pattern over the entire surface of the cell. The cell has short anterior and long posterior flagella (Fig. 1g–h, 8a–c). The anterior flagellum is inserted transversally and no undulation of either flagellum is observed. Both flagella are covered by elliptical scales along their entire length (Fig. 8d). The scales are c.a. 120 nm in length and 100 nm in width. The scales are arranged in longitudinal rows. In each row, the scale proximal to the flagellar insertion overlaps the distal one. Distance between the centre of the neighbouring scales is ca. 100 nm. The posterior flagellum is ca. 380 µm in diameter, along the proximal half of the length, then bluntly reduce to ca. 250 µm in diameter along the distal half. The anterior flagella is consistent in its diameter, ca. 250 µm. Mastigonemes are also present on the entire anterior flagellum, and the distal half of the posterior flagellum bears fine hairs. (Fig. 8e).

### Molecular Phylogeny of *Psammosa* spp

**Figure 9:**
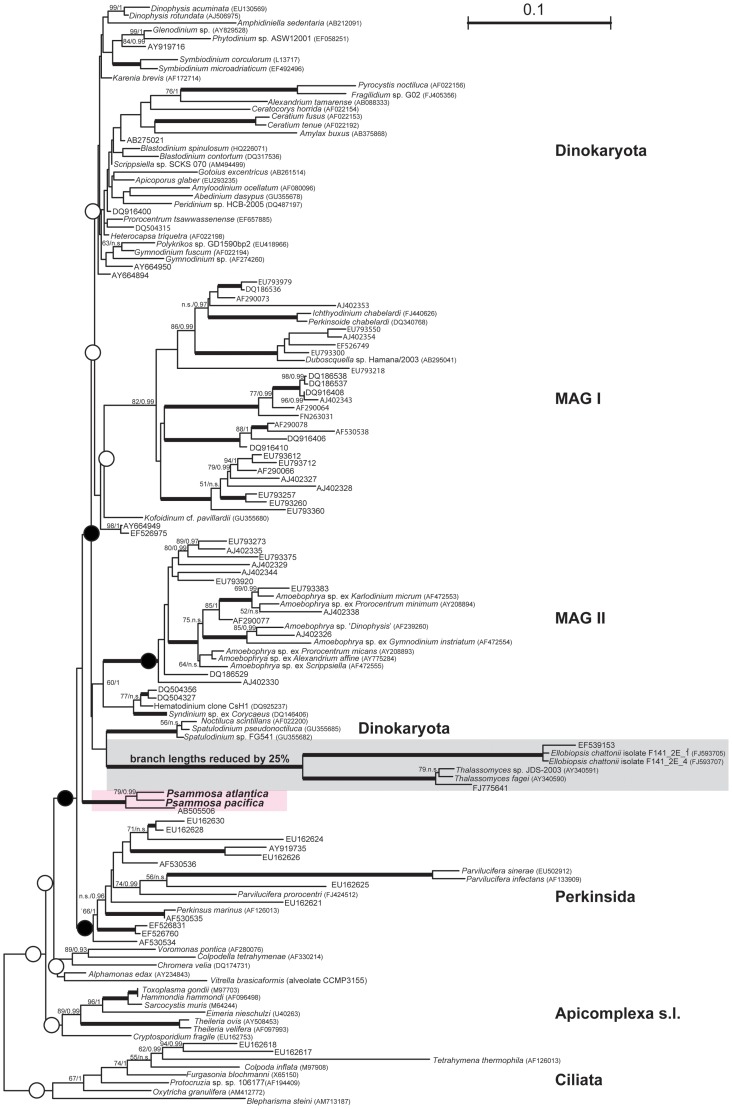
Small subunit ribosomal RNA (SSU rRNA) maximum likelihood phylogeny (ML) of two *Psammosa* species in context of recently described biodiversity of perkinsids and ‘lower’ dinoflagellates. The tree was inferred using RAxML 7.2.8 under the GTR model with gamma correction from at total 1297 nucleotides. Numbers at nodes represent branching support. First number shows ML non-parametric bootstrap support as inferred in RAxML (500 replicates). Second number represents bayesian inference posterior probability, as calculated using Phylobayes 3.2 under the empirical admixture model C40 combined with LG exchange rate matrix. Only posterior probability higher than 0.94 and bootstrap support of 50 and more is shown. Branch lengths of ellobipsids (shaded clade) were reduced as indicated on figure. Dots by nodes point to alternative topologies of *Psammosa* spp. as tested by AU test (empty represent rejected and black not-rejected). Uncultured/environmental sequences are represented only by GenBank accession number. Accession numbers of sequences with known taxonomy are shown in brackets. See text for more details.

Bayesian phylogeny based on SSU rRNA (Fig. 9) recovered the main lineages as expected: (1) MAG I, which includes *Euduboscquella* and *Ichthyodinium*; (2) MAG II, which includes *Syndinium*, *Hematodinium* and *Amoebophrya*; (3) the ellobiopsids clade, which includes *Ellobiopsis* and *Thalassomyces*; and (4) the dinokaryotes. Although *Oxyrrhis marina* is a taxon of interest for this part of the tree, it was excluded from analysis due to its notoriously long branch. It was confirmed overall tree topology did not change significantly whether it was included or excluded (not shown). The *Psammosa* lineage in this tree branches independently of any other major group at the base of whole dinoflagellate lineage (including perkinsids and syndineans *s.l.*), though without any support. An environmental sequence (AB505506) from the deep sea [55] was also found to fall within the *Psammosa* lineage. Maximum likelihood (ML) analysis yielded somewhat different topology with *Psammosa* lineage branching between perkinsids and syndineans (as indicated by red arrow on Fig. 9). We performed approximately unbiased (AU) test [56] to discriminate between these two hypotheses. As expected, both couldn’t be rejected on 0.05 significance level. In order to determine whether there are other possibly non-rejected topologies of *Psammosa* spp., we forced the monophyly of *Psammosa* sequences to all of the major clades (ciliates, apicomplexans, perkinsids, MAGI, MAGII, ellobiopsids and dinokaryotes), re-optimized the maximum likelihood (ML) topologies using RAxML and performed additional AU test. Results reveal there is one alternative non-rejected topology with *Psammosa* spp. branching at the base of the *Amoebophrya* (MAGII) clade (see Fig. 9 for visualization).

**Figure 10:**
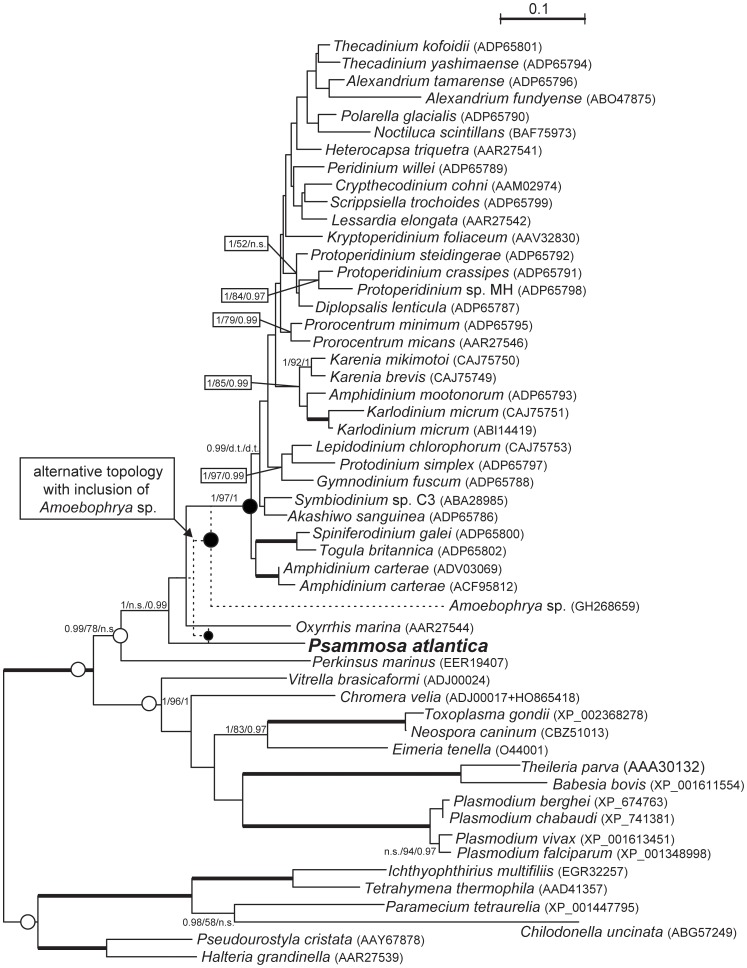
Phylogenetic position of *Psammosa atlantica* as revealed by maximum likelihood (ML) analysis of amino acid sequences of Hsp90 gene (532 aa included). The tree was inferred using PhyML-CAT software under the empirical admixture model C40. Numbers at nodes represent branching support with the first number being result of a approximate likelihood-ratio test (a-LRT) computed in PhyML-CAT (under the above specified model). The second number shows bayesian posterior probability as inferred with Phylobayes 3.2 software under the empirical admixture model C40 combined with LG exchange rate matrix. The third number represents non-parametric bootstrap support of maximum likelihood (ML) analysis as inferred by RAxML (LG matrix, 500 replicates). Posterior probability and a-LRT higher than 0.94 and bootstrap support of 50 and more is shown. Dotted lines reveal ML and BI topology of alternative Hsp90 dataset with syndinian *Amoebophrya* sp. included. Dots by nodes point to alternative topologies of *Psammosa* spp. as tested by AU test (empty represent rejected and black not-rejected). Uncultured/environmental sequences are represented only by GenBank accession number. Accession numbers of sequences with known taxonomy are shown in brackets. See text for more details.

In bayesian phylogenies based on Hsp90 (Fig. 10), *Psammosa atlantica* branched between *Perkinsus marinus* and *O*. *marina*, but again without significant support (i.e. posterior probability 0.95 and higher and bootstrap support over 50). Maximum likelihood analysis yielded somehow different topology with *P. atlantica* and *O*. *marina* forming clade at the base of dinokaryotes (see dashed topology at Fig. 10). Neither hypotheses are rejected by AU test. Similarly to SSU dataset, we have also created alternative topologies by ML and tested them again. To our surprise, another topology that was not rejected was the one with. *P*. *atlantica* branching at the base of apicomplexan s.l. clade (i.e. including colpodellids and chromerids), although with marginal probability (0.063).

We also have created alternative dataset including partial sequence of *Amoebophrya* sp. ex *Karlodinium veneficum*. Here, the *P*. *atlantica* again forms clade with *O*. *marina* and branch between *P*. *marinus* and *Amoebophrya* (dotted topology at Fig. 10). The post-perkinsid origin of *Psammosa* lineage is further supported by deletion of an amino acid in Hsp90 that are characteristic of dinoflagellates and *O. marina*, but are absent in other eukaryotes including *P. marinus* and apicomplexans.

## Discussion

### Novelty and Distribution of *Psammosa*

In this study, we reported two new species of a novel lineage of early dinoflagellates, *Psammosa pacifica* and *P. atlantica* from an intertidal sandy beach environment. Based on their unique phylogenetic position and morphology, we have erected a new genus for these two species: *P. pacifica* and *P. atlantica*. They shares general cell structure, i.e. compressed barley shape body with a subapical diagonal ridge, while the difference in cell length (i.e., *P. pacifica* is 7–8 µm; *P. atlantica* is 10–13 µm) and the contour of the posterior part of the cell (*P. pacifica* has acute end; *P. atlantica* is truncated end). In addition to the morphological difference, their prey preferences are different. *P. atlantica* feeds on *Navicula* sp., while *P. pacifica* does not feeds on *Navicula* sp. or other diatoms, but on *Spumera* sp. Molecular sequences of ribosomal SSU are not identical between two species. Based on those similarity and dissimilarity, we concluded that two organisms are members of the same genus *Psammosa*, but different species.

Although compressed barley shaped cell with laterally inserted flagella is not necessarily defining characters among protists, the centrally located protrusion and cell architecture separated into right anterior and left posterior parts, presence of the refractile body and two flagella inserted in two separate grooves delineate the genus *Psammosa* from non-alveolate colourless flagellated protists such as katablepharids (lacking protrusion and refractile body; both flagella emerge from a single depression; possessing light microscopically recognizable ejectisomes) or developayellids (lacking protrusion and refractile body; both flagella emerge from a single depression; bacterivores).

Some alveolate flagellate especially colpodellids are light microscopically similar to *Psammosa*. In fact we found one species, *Colpodella unguis* resembles most to *Psammosa*. *Colpodella unguis* is originally described solely based on light microscopy from the shallow sandy sediment of Shark Bay, Western Australia, though its placement to genus *Colpodella* is tentative [57]. Light micrographs from the original description of *C. unguis* share some similar characteristics with *Psammosa pacifica*; specifically, (1) two laterally inserted mastikonts, and (2) elongated reniform cell of similar size range (7–8 µm for *P. pacifica*; 7–10 µm for *C. unguis*) with a protrusion in the middle. However, the proportion of anterior left part is different: the left anterior part of *P. pacifica* is about two thirds of the cell length, whereas that of *C. unguis* is about one third, and thus forms an acute end described as “rostrum” in the original description [57].

Interestingly, Myl’nikov [58] re-isolated *C. unguis* from a different locality and performed ultrastructural observations, which also revealed a similar transition plate (though lacking the proximal dark stained inclusion body we observed), and an apical complex composed of a pseudoconoid (though with more than eight microtubules) as well as ‘micronemes’ and ‘rhoptries’ (see discussion below). The nuclear morphology of the Russian *C. unguis* is also distinct from that of *P. pacifica* in that it is characterized by a fibrous nuclear content, which may be due to different preparation conditions. The russian strain of *C. unguis* is also shown to proliferates via “oblique-transversal” cell division, rather than via cyst divelopment. Based on the light microscopical features, its habitat, life history and ultrastructures, it is possible that *C. unguis* may well be another member of the lineage. It is interesting the correct position of *C. unguis* in relation to *Psammosa* spp. In this study, however, we don’t have any direct evidence of their correct phylogenetic position, such as SSU rDNA sequence. Thus, we will refrain from proposing any taxonomic changes to *C. unguis*.

### Habitat and Trophic Strategy

Both species of *Psammosa* from different location opposite side of the continent but under very similar condition, i.e., in the top interstitious layer of a dissipative beach. *Colpodella unguis*, the suspected member of this lineage is also found from shallow marine benthic habitat [57], [58], [59]. This newly recognised lineage would be associated to interstitious/benthic habitat and is not included in the water column. This would explain why there is only one environmental sequence that belongs to the *Psammosa* clade. We recognised a previously unidentified environmental sequence from coastal marine sediment of about 1000 m depth [55] that is also a member of the *Psammosa* clade, although it is unclear if the sequence is from an actively growing cell, or a dormant cyst that drifted into the sediments [60]. The revolution of our knowledge of protist diversity and distribution has been restricted to particular environments such as coastal marine water [19], [24], [28], open ocean [19], [24], [28], [29], deep sea beds [20], [21], [24], [26], anoxic/oxygen deplete marine environments [18], [22], [23], [24], [30], arctic ocean [24], [25], or freshwater [24], [61]. Although our view of protist biodiversity has been greatly improved by those environmental survey, there are different environments to be investigated, and marine intertidal sandy beach is one of the candidate environment, considering previous conventional surveys and the frequency of novel taxa discoveries.

*Psammosa* spp. are heterotrophic eukaryvores, while all the basal dinoflagellates and the sister group, perkinsids are parasitic, except *Oxyrrhis marina* that is also a heterotrophic eukaryvore. Considering perkinsids, the immediate outgroup of *Psammosa*, are also parasitic, it may appear predation would be extraordinary cases. However, it may also be the case that only parasitic lineages have been described so far and that vast majority of diversity demonstrated by environmental surveys may include organisms of various trophic strategies. It is not necessarily appropriate to assume that MAG lineages to be all parasitic, considering the diversity included in those clades are somewhat equivalent to that of the dinokaryotes, which include various trophic modes ranging from photosynthesis, predation, symbiosis and parasitism. Cellular level investigation of unobserved MAG I and MAG II, as well as yet to be discovered novel lineages will be required to fill the gap of our knowledge.

### Molecular Phylogeny

Molecular phylogeny based on small subunit ribosomal RNA (SSU rRNA) and Hsp90 recovered monophyly of dinoflagellates and perkinsids, but the branching order within the clade is not resolved with any support by bootstrap value or Bayesian post probability. Although topologically equivalent, the support of those branching is lower than some of the previous studies [32], [33], [34], [35], [36], [37], [38], [39], [44], [61], [62], [63], [64], [65]. This may be partly because our trees retain more taxa including fast evolving clades, such as the ellobiopsids in SSU rRNA tree, which artifactually attracts noctilucoid clades in the ML tree.

**Figure 11:**
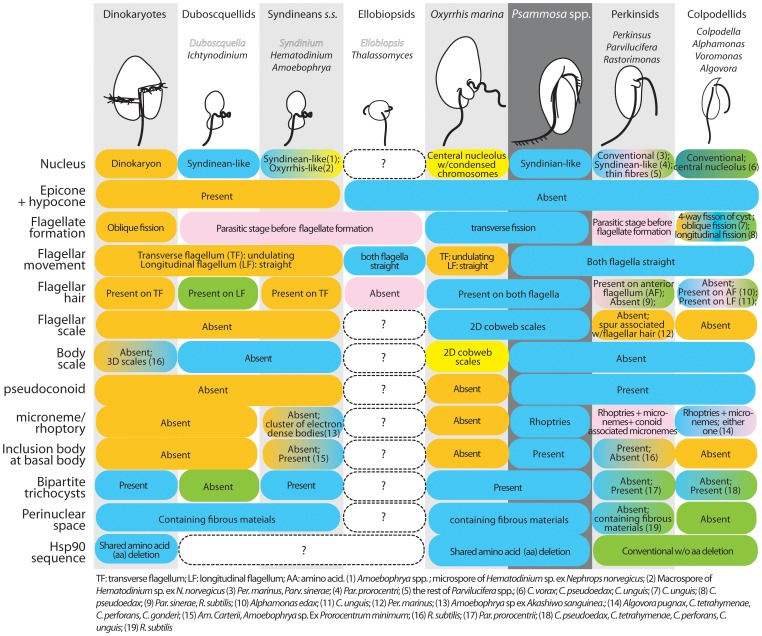
Character matrix of *Psammosa* and the flagellate stage of dinoflagellate lineages, perkinsids and colpodellids.

The best ML trees of SSU rRNA placed *Psammosa* either basal to dinoflagellates after divergence of perkinsids. AU test also did not exclude the possibility that (b) *Psammosa* branches from common ancestor of perkinsids and dinoflagellates, or (c) *Psammosa* is basal to *Amoebophrya* clade within MAG II (never observed as the best topology). In Hsp90 tree, topology (b) was consistently rejected; instead, topology (a) was supported regardless whether with or without a short Hsp90 fragment of *Amoebophrya* in the analyses. The inconsistency among Hsp90 analyses is in the affinity between *Psammosa* and *O. marina*; *Psammosa* branches after divergence of Perkinsids but before that of *O. marina* in a Bayesian tree without a short *Amoebophrya* sequence, whereas they form a monophyletic clade of a Bayesian tree that includes partial *Amoebophrya* sequence and a ML tree. As both sequences of *P. atlantica* and *O. marina* are rather divergent and their relationship can be a result of so called long-branch artifact. This close affinity between *Psammosa* and *O. marina* is consistent with the ultrastructural and other characters shared between two, as we discuss below (Fig. 11).

Unfortunately, due to extreme nature of *O. marina* SSU rRNA sequence, we are unable to include this key organism in this widely used and reliable phylogenetic marker. In comparison, although the evolutionary rate of *O. marina* and other taxa is modest in Hsp90 trees, the taxon sampling in Hsp90 trees is not as thorough as SSU rRNA trees.

Resolving this part of tree is one of the urgent agenda. Although increased number of sequence data are registered in genbank, most of them are sequences of ITS region of SSU rRNA, which are not informative for resolving deep branching that we focus on here. A small EST library of *Amoebophrya* sp. includes ribosomal proteins in addition to a fragment of Hsp90 (included in our analyses), which is potentially useful in future though requires the same set of gene sequences from the related taxa. We are currently conducting expressed sequence tag (EST) analyses of *P. pacifica.* Currently there are publicly available EST libraries of *O. marina* and *P. marinus*. With an addition of *P. pacifica* EST data, along with ongoing EST projects of other dinoflagellates and other basal dinoflagellate taxa, we will have a better insight to the early evolution of the dinoflagellates.

Due to the uncertainty of its phylogenetic position in relation to the other lineages of dinoflagellates and perkinsids, we refrain to raise any higher taxa for *Psammosa* spp. to avoid unnecessary confusion.

### *Psammosa* Retains Ancestral Characteristics of Early Dinoflagellates

Morphologically, both *Psammosa* species shares various characters not only with dinoflagellates but also with perkinsids and colpodellids which are thought to have retained a close resemblance to the ancestor of apicomplexans and dinoflagellates [2], than they do dinokaryotes.

The dinokaryotes are by far the most studied group of dinoflagellates, and are widely acknowledged to have very unique morphological characters, such as dinokaryon, the cell with epicone and hypocone, the undulating tranverse flagellum [7], [49], as well as a variety of unusual molecular and genomic characteristics such as mitochondrial RNA editing, plastid mini-circle genome, and the addition of spliced-leaders to cytosolic transcripts [8], [9], [10], [11], [12], [13], [14]. Those features must have been acquired relatively early in the evolution of the dinoflagellates, but many are missing from the, perkinsids, suggesting that the the order of some steps in this evolution may yet be elucidated by examining the state of early-branching lineages such as *Psammosa*, *Oxyrrhis*, and syndineans for possible intermediates [62], [63].

Morphologically *Psammosa* shows interesting mosaïc of the characters shared with dinokaryotes, syndineans and perkinsids. As is often the case with dinokaryotes in particular and parasite in general, the related lineages of *Psammosa* have developed unique morphological and lifecycle characters, which sets a challenge for inferring homology of characters and their evolution. Here in this study, our focus is newly described flagellate genus *Psammosa*, hence we limit our discussion to the flagellate stages, where non-flagellated stages are also known. Fig. 11 summarizes characters of dinokaryotes, syndineans, *O. marina*, *Psammosa* spp., perkinsids and heterotrophic colpodellids. *Psammosa* shares characters found in dinokaryotes, namely, presence of bipartite trichocysts [66] and perinuclear space filled with fibrous materials, which have been interpreted as the precursor of flagellar hair by several authors based on morphological resemblance, though without chemical or molecular evidence [53], [67], [68], [69]. These characters are not synapomorphy of dinoflagellates. Bipartite trichocyst is acquired in the common ancestor of dinoflagellates, perkinsids and colpodellids, as it is also found in *Parvilucifera prorocentrii* [70] and several *Colpodella* spp. [58], [71], [72]; while the perinuclear space with fibrous materials is a synapomorphy of dinoflagellates and perkinsids as it is found in *Rastorimonas subtilis* [73].

*Psammosa* shares characters with the flagellates stage of syndinians, such as “syndinean-like” nucleus found in *Ichtyodinium chabelardi* [44], *Amoebophrya* spp [51], [52], the microspore of *Hematodinium* sp. Ex *Nephros norvegicus* [50] and a perkinsid *P. prorocentrii* [55]. Also, the inclusion body (an electron dense globule) at basal body are found in *Psammosa*, *Amoebophrya* spp. [51], [52], *Parvilucifera* spp. [74], [75] and *R. subtilis* [73]. These characters is most likely acquired in the common ancestor of dinoflagellates and perkinsids.

As the close affinity was suggested in molecular phylogeny, *Psammosa* resembles to *O. marina* in many regards. *Psammosa* has two dimensional cobweb type flagellar scale. It is known that *O. marina* has the same type of scales on the flagella and the cell surface [59]. Two dimensional cobweb scales are only found in *Psammosa* and *O. marina* [76], as opposed to three dimensional body scales that have been reported in some dinokaryotes, i.e., Heterocapsa spp. [77], Lepidodinium spp. [78], [79]; Amphidinium cupulatisquamata [80]. In addition to flagellar scales, *Psammosa* and *O. marina* also bear flagellar hair on both anterior and posterior flagella. These characters are only restricted to these two genera. *Psammosa* and *O. marina* also share the mode of proliferation, i.e., a flagellate mother cell divides binary along the transverse plane and directly form two daughter flagellated cells [81]. This division would be homologous to the oblique cell division of dinokayrotes [82]. *Colpodella unguis* is also reported to perform “oblique-transversal” cell division [58], in contrast to majority of other known colpodellids undergo cyst development prior to four-way cell division; as well as the curved basal plate in their flagellar transition region [58], in contrast to a flat plate found in the flagellar transition region of *O. marina* [69], perkinsids [75], [83], [84], [85] or other colpodellids [58], [72], [73], [86], [87]. However, the placement of this species in *Colpodella* is “tentative” and has not been justified by molecular as was discussed earlier, we refrain from drawing a strong conclusion from the timing of acquisition of these characters.

### Presence of the Apical Complex and Character Evolution of the Dinoflagellates

The most intriguing characteristic that *P. pacifica* retains is the apical complex. It is widely accepted that the apical complex was acquired in the common ancestor of myzozoans (dinoflagellates, perkinsids, colpodellids, and apicomplexans), then during dinoflagellate evolution was secondarily lost or altered to such an extent that it is no longer recognizably homologous [2]. Perkinsids also have an apical complex, but this is the first detailed evidence for an apical complex in the (?lineages branching after perkinsids?) dinoflagellates (Gestal et al [44] mention the possible presence of pseudoconoid in the syndinian *Icthyodinium chabelardi*, but the structure referred to is more likely a part of flagellar root apparatus) and the complex found in *P. pacifica* is generally simpler than that of perkinsids. One unique aspect of the apical complex of *P. pacifica* is that it has a gullet, or a narrow invagination associated to the apical complex (see Fig. 7a–b), which is absent in perkinsids, but might be related to the apical pore found in some dinokaryotes [87], [88]. In perkinsids, the apical complex typically consists of a pseudoconoid (discussed below) and two or more dark stained membrane-bounded structures directly associated to the pseudoconoid [70], [74], [75], [83], [84]. Those structures are referred to as either “rhoptries” or “micronemes” or some derivative, based on their similar appearance to those structures in apicomplexans, though there is as yet no direct evidence of homology. *Psammosa pacifica* possesses elongated rhomboid structures that are directly associated with the pseudoconoid, and dark stained vesicles that are posterior and relatively distant from the pseudoconoid. We have chosen to refer the elongated rhomboid structure of *P. pacifica* as a “rhoptry” based on its appearance and association with the pseudoconoid, though again its homology to rhoptries in other myzozoans must be tested. The profile of the pseudoconoid in *P. pacifica* typically shows eight microtubules, though there seems to be additional microtubules running posteriorly towards the basal bodies (data not shown). This would suggest another parallel with *Perkinsus marinus*, where some of pseudoconoid microtubules extend to the posterior [89], but to address this question, and the overall state of this interesting structure, a detailed three dimensional reconstruction of the apical complex would be required.

Interestingly, a recent ultrastructural investigation of *Amoebophrya* sp. from *Akashiwo sanguinea* revealed the presence of elongated “electron-dense bodies” in the anterior part of the infectious flagellate cell [53] that seemingly used for host invasion, similar to the rhoptries and micronemes of the apicomplexan parasites, [90]. It will be interesting to investigate the apical complex of *Psammosa* and possibly “electron-dense bodies” of *Amoebophrya* to the well-studied apicomplesans’ apical complex in detail, e.g. three dimensional architecture or molecular sequence level to test the possible homology and evolutionary link.

### Concluding Remarks

In this study, we report a new lineage of early-diverging dinoflagellates, which we name *Psammosa*, with description of two new species of *P. pacifica* and *P. atlantica*. *Psammosa* displays a range of interesting morphological characteristics further supporting its divergence early in the evolution of dinoflagellates. Specifically, it retains a number of characters likely to be ancestral to the group, most importantly the apical complex, which is common among the apicomplexans, colpodellids and perkinsids but never before observed in dinoflagellates. Based on its potential to illuminate the origin of a number of strange and unique characteristics that appeared early in the evolution of apicomplexans and dinoflagellates, further study of the *Psammosa* apical complex and its genomics and gene expression systems seem likely be of particular interest.

## Materials and Methods

### Collection and Culture Conditions

No specific permits were required for the described field studies. *Psammosa pacifica* was isolated from a sand sample collected at Boundary Bay, British Columbia, Canada (49.00863°N; −123.02281°W) on 15th April 2010. *Psammosa atlantica* was isolated from a sand sample collected at Blomidon Beach in the Bay of Fundy, Nova Scotia, Canada (45.25580°N; −64.34907°W) on 30th July 2008. Notably, both beaches are characterised by an extremely flat beach face due to the macrotidal range and to the very sheltered nature of the beach, respectably. The surface layer of wet sand was collected from the intertidal zone, samples vigorously shaken with K-Si medium [91], and suspensions incubated at 18°C under the cycle of Light:Dark  = 6 h:18 h. Subsequently, single cells were isolated by micropipetting. *Psammosa pacifica* was incubated in modified K-Si medium with addition of 1 ml of 95% ethanol saturated with ubiquinone per 1000 ml, with a strain of bacterivorous stramenopile (Tofino-D6Ga) and *P. atlantica* was incubated with *Navicula* sp. (PRA-314, ATCC, VA) as a food source. Prey organisms were pre-cultured separately either in a polystyrene culture plate or a polystyrene culture flasks. *Psammosa* spp. were added whenever the prey cells were fully consumed in the previous inoculation. The *P. atlantica* strain was no longer viable in culture after 6 months.

### Microscopy

Live cells were observed using differential interference contrast (DIC) microscopy using an Axioplan2 compound microscope (Zeiss, Germany) equipped with an XL H1s camcorder (Cannon, Japan) mounted using a PROHDVC adaptor (Micro Tech Lab, Austria) with an additional 6 mm ring.

Scanning electron microscopy (SEM) was carried out by fixing cell cultures of P. pacifica and P. atlantica in K-Si medium containing 2.5% Glutaraldehyde (final concentration) on coverslips coated with poly-L-lysine or polyethyleneimine at room temperature for 30 min. The cells were rinsed three times with distilled water, then dehydrated through a graded series of ethanol and critical point dried with CO2 using a Tousimis Samdri 795 CPD (Rockville, MD). Dried coverslips were mounted on aluminum stabs and then sputter coated with gold (5 nm thickness) using a Cressington high-resolution sputter coater (Cressington Scientific Instruments Ltd, Watford, UK). The coated cells were viewed under a Hitachi S4700 scanning electron microscope (Hitachi, Japan).

Whole mount transmission electron microscopy (TEM) was performed using actively growing *Psammosa pacifica* culture isolated by micropipetting, fixed with 2% Uranyl acetate (final concentration) for 5 mins on a formvar coated mesh grid and rinsed with distilled water. The grid was viewed under a Hitahci H7600 transmission electron microscope (Hitachi, Japan).

Serial ultrathin and thin section TEM was also performed on actively growing *Psammosa pacifica* culture harvested by centrifugation at 1000 *g* for 15 minutes that was semi-simultaniously fixed [92] with 2.5% Gutarardehyde and with 0.01% Osmium tetroxide in sea water (final concentration, respectively) for 1 hour at room temperature. Cells were then rinsed once with distilled water, dehydrated through ethanol series, then embedded in SciPon resin in beam capsules. Serial ultrathin sections (50 nm thickness) were collected on Formvar-coated slot grids. Ultrathin sections were post stained with uranyl acetate for 15 minutes and lead citrate for 5 minutes [93]; then observed under a Hitachi H7600 electron microscope (Hitach, Japan), and post processed on a Photoshop CS5 software (Adobe, CA).

### Molecular Methods and Phylogenetic Analyses

100 cells of *P. pacifica* and *P. atlantica* were isolated using micropipette to avoid the contamination of the prey cells. DNA samples were prepared from the manually isolated cells using MasterPureTM Complete DNA & RNA Purification Kit (Epicentre Biotechnologies, WI). Small subunit (SSU) rRNA and Hsp90 genes were amplified by nested PCR using primers listed in table S1. PCR reactions were performed following Okamoto et al for SSU rRNA [94] and Kim et al for Hsp90 [95]. Although template DNA has a minimum contamination of the prey cells, sequences were determined after subcloning of PCR products to avoid the possible contamination. Sequences were deposited in Genebank under accessions (SSU of *P. pacifica*: JN873311; SSU of *P. atlantica*: JN873310; Hsp90 of *P. atlantica*: JN873312).

Sequences of *Psammosa* were aligned with other alveolate sequences using arb-aligner (http://www.arb-silva.de/aligner/) for SSU rRNA and Mafft 6.86 [96], [97] for Hsp90. Ambiguous parts of alignments were deleted using SeaView 4 [98]. Maximum likelihood topology was calculated using RAxML 7.2.8 [99] under the GTR (SSU rRNA) and LG (Hsp90) models of evolution. The phylogeny with highest likelihood score was chosen from 200 independent runs each starting with different starting topology. Non-parametric bootstrap support was estimated from 500 replicates. Bayesian posterior probabilities were calculated using Phylobayes 3 [100] under the CAT admixture model (limited to 40 rate categories) combined with GTR (SSU rRNA) and/or LG (Hsp90) exchange rates. Aproximately unbiased topology test was performed using Consel [101].

### Nomenclatural Acts

The electronic version of this document does not represent a published work according to the International Code of Zoological Nomenclature (ICZN), and hence the nomenclatural acts contained in the electronic version are not available under that Code from the electronic edition. Therefore, a separate edition of this document was produced by a method that assures numerous identical and durable copies, and those copies were simultaneously obtainable (from the publication date noted on the first page of this article) for the purpose of providing a public and permanent scientific record, in accordance with Article 8.1 of the Code. The separate print-only edition is available on request from PLoS by sending a request to PLoS ONE, Public Library of Science, 1160 Battery Street, Suite 100, San Francisco, CA 94111, USA along with a check for $10 (to cover printing and postage) payable to “Public Library of Science”.

In addition, this published work and the nomenclatural acts it contains have been registered in ZooBank, the proposed online registration system for the ICZN. The ZooBank LSIDs (Life Science Identifiers) can be resolved and the associated information viewed through any standard web browser by appending the LSID to the prefix “http://zoobank.org/”. The LSID for this publication is: urn:lsid:zoobank.org:pub:D2BDEF96-E795-47508BB1-4D213DFC952A.

## Supporting Information

Table S1 **List of primers used in this study.**
(DOC)

Movie S1 **Movie clip showing** ***Psammosa pacifica*** **resting on a bottom surface.**
(MP4)

Movie S2 **Movie clip showing swimming behaviour of** ***Psammosa pacifica*** **at low magnification.** *Psammosa* switches between two modes of swimming, namely, “swimming with rotation” and “spiraling without rotation”.
(MP4)

Movie S3 **Movie clip showing** ***Psammosa pacifica*** **swimming at high magnification.** The cell is before division, with posterior flagella duplicated.
(MP4)
