# Supplementary material for: Description of Two Species of Early Branching Dinoflagellates, Psammosa pacifica n. g., n. sp. and P. atlantica n. sp
Source: PLoS One. 2012 Jun 18;7(6):e34900. doi: 10.1371/journal.pone.0034900 (PMC3377698; doi:10.1371/journal.pone.0034900)
Supplement: Table S1 — List of primers used in this study. (DOC) [file pone.0034900.s001.doc]

**Supplemental table 1. Primers used in this study.**

Primers used in this study and references are listed below. S: sense direction; AS: antisense direction.

| Primer | direction | Sequence | Reference |
| --- | --- | --- | --- |
| SSU |  |  |  |
| NPF1 | S | 5'-TGCGCTACCTGGTTGATCC-3' | Chantangsi and Leander (2009) |
| 525F | S | 5'-AAGTCTGGTGCCAGCAGCC-3' | Hoppenrath et al 2007 |
| 1050MRD | AS | 5'-GCCTYGCGACCATACTCC-3' | Chantangsi and Leander (2009) |
| FAD | AS | 5'-TGATCCTTCTGCAGGTTCACCTAC-3' | ‘B’ in Medlin et al 1998 |
| Hsp90 |  |  |  |
| 100XF | S | 5'-CAG CTG ATG TCC CTG ATC ATY AAY CAN TTY TA-3' | Simpson et al (2002) |
| HspFC | S | 5'-TSA AGG ACC TSR TCA AGA AGC A-3' | Kim et al (2006) |
| HspRD | AS | 5'-CTC NCC RGT GAT GWA GTA GAT-3' | Kim et al (2006) |
| 910XR | AS | 5'-TCG GGG TTG ATY TCC ATN GTY TT-3' | Simpson et al (2006) |

Chantangsi, C, Leander BS (2009) An SSU rDNA barcoding approach to the diversity of marine interstitial cercozoans, including descriptions of four new genera and nine new species. Int J Syst Evol Microbiol *in review*

Moreira D, von der Heyden S, Bass D, Lopez-Garcia P, Chao E, Cavalier-Smith T. 2007. Global eukaryote phylogeny: Combined small- and large-subunit ribosomal DNA trees support monophyly of Rhizaria, Retaria and Excavata. Mol Phylogenet Evol. 44:255-266.

Hoppenrath M, Horiguchi T, Miyoshi Y, Selina M, Taylor MFJR, Leander BS (2007) Taxonomy, phylogeny, biogeography, and ecology of *Sabulodinium undulatum* (Dinophyceae), including an emended description of the species. Phycol Res. 55(2):159–175

Medlin L, Elwood HJ, Stickel S, Sogin ML. (1988) The characterization of enzymatically amplified eukaryotic 16S-like rRNA-coding regions. Gene 71:491-499.

Simpson AGB, Lukes J, Roger AJ. 2002. The evolutionary history of kinetoplastids and their kinetoplasts. Mol Biol Evol. 19:2071–2083.

Simpson AGB, Inagaki Y, Roger AJ. 2006. Comprehensive multi-gene phylogenies of excavate protists reveal the evolutionary positions of “primitive” eukaryotes. Mol Biol Evol. 23: 615–625.
